# Shigella as a Cause of Diarrhea Hospitalization in Children Under Five: Evaluation by Conventional and Molecular Methods

**DOI:** 10.7759/cureus.50546

**Published:** 2023-12-14

**Authors:** Pallavi Bansal, Dheeraj Shah, Rajesh Kumar Meena, Gargi Rai, Shukla Das, Manish Narang, Piyush Gupta

**Affiliations:** 1 Pediatrics, University College of Medical Sciences, Delhi, IND; 2 Microbiology, University College of Medical Sciences, Delhi, IND; 3 Microbiology, University College of Medical Sciences, New Delhi, IND

**Keywords:** under-five children, under-five diarrhea, acute infectious diarrhea, shigella, stool culture, polymerase chain reaction, dysentery, diagnosis, children

## Abstract

Background and objectives: *Shigella *is an important cause of diarrhea in children under five, often missed by conventional laboratory methods. Blood in stools has always been a syndromic indicator for *Shigella *diarrhea, but most cases present with watery diarrhea without blood. This study aimed to determine the frequency of *Shigella *detected by molecular and conventional methods in children under five. Additionally, we aimed to study the clinical profile and outcome of children with *Shigella *diarrhea managed as per current diarrhea treatment guidelines.

Methods: In this hospital-based prospective observational study, stool samples from 150 children (age range: one month to five years) with acute diarrhea (duration < seven days) were subjected to routine microscopic examination, stool culture, and DNA extraction. The extracted DNA from stored stool samples was subjected to polymerase chain reaction (PCR) amplification using a specific primer for the invasion plasmid antigen H gene sequence (ipaH) gene at 424 bp. Results were interpreted in the context of the percentage of isolation of *Shigella *by molecular (PCR) and conventional methods (stool microscopy and culture) and the follow-up outcome in terms of recurrence of diarrhea or dysentery and growth faltering over three months after discharge.

Results: *Shigella *infection was diagnosed in stool samples by PCR from 13 (8.7%) children, whereas it was isolated by conventional stool culture in only one (0.7%) child. The sensitivity of culture was only 7.7% against PCR for the diagnosis of *Shigella *infection, whereas blood in stools had a sensitivity of 15.4%. The majority of *Shigella *PCR-positive cases (11 out of 13) presented with non-bloody diarrhea. None of the evaluated clinical predictors had a significant association with the *Shigella *infection. No statistically significant difference was found between PCR-positive and PCR-negative children at the end of follow-up (P>0.05).

Conclusion: The majority of children with *Shigella *infection present with watery diarrhea rather than bloody diarrhea, and a history of blood in stools is a poor marker for the diagnosis of shigellosis. The diagnostic performance of stool culture is also very low compared to stool PCR for the diagnosis of *Shigella *diarrhea.

## Introduction

Diarrhea is one of the top three infectious diseases responsible for ~8%-10% of total deaths among children below the age of five years; 90% of these deaths are estimated to occur in South Asia and sub-Saharan Africa [1, 2]. Rotavirus and diarrheagenic *Escherichia coli* (*E. coli*) are the two most common pathogens responsible for acute diarrhea in children below the age of five years. *Shigella*, *Cryptosporidium*, *Campylobacter spp.*, and *Vibrio cholerae* are a few other pathogens frequently associated with diarrhea in children under five [3], with variable region-specific incidence.

The Global Enteric Multicentric Study (GEMS), a large case-control study, determined the etiology of childhood diarrhea in South Asia and sub-Saharan Africa. Rotavirus, *Cryptosporidium*, *E. coli* producing heat-stable toxin (ST-ETEC), and *Shigella *were identified as major attributable organisms for the cause of diarrhea [4]. Later, stool samples from GEMS were re-analyzed using quantitative polymerase chain reaction (PCR) and revised pathogen-specific burdens were calculated. Pathogen-attributable burden increased significantly, and *Shigella *was reported as the most common causative agent [5]. Customarily, *Shigella *infection is associated with bloody diarrhea, but this re-analysis using PCR techniques documented that most such children have only loose stools without blood. Despite *Shigella *being reported as the most common cause of acute diarrhea in GEMS re-analysis, there is a paucity of studies evaluating *Shigella *by molecular methods and comparing the isolation rate with conventional culture techniques. Also, the clinical characteristics and outcomes of children with non-bloody *Shigella *diarrhea managed as per current WHO diarrhea treatment guidelines are not known.

In this study, we determined the frequency of *Shigella *detected by molecular and conventional methods in under-five children with diarrhea and the clinical profile and outcome of children with *Shigella *diarrhea over the next three months.

## Materials and methods

This hospital-based prospective observational study was conducted between November 2017 and April 2019 at the University College of Medical Sciences, Delhi, an urban hospital catering mainly to the low-income population of eastern Delhi and adjoining areas of the state of Uttar Pradesh in India. Approval from the Institutional Ethics Committee for Human Research (IEC-HR) of the institute was obtained before enrolling the study participants (approval number: IEC-HR/2017/32/88). Informed consent was obtained from the parents or caregivers of all the participants enrolled in the study.

Participants

All consecutive children aged between one month and five years, presenting with acute diarrhea to the outpatient department or pediatric emergency services of the hospital and requiring hospitalization due to any reason (such as dehydration, severe malnutrition, septicemia, etc.), were eligible for inclusion. Acute diarrhea was defined as diarrhea of < seven days duration with or without blood in stools [6]. Children who received antibiotic therapy seven days prior and those with any other known cause of blood in stools (rectal polyp, bleeding diathesis, inflammatory bowel disease, etc.) were excluded.

At the time of enrollment, detailed clinical history, including the treatment prescribed (oral rehydration solution (ORS), zinc, paracetamol, etc.) during the current diarrheal episode, was elicited from the parents or guardian. A detailed clinical examination, including an assessment of hydration status, was performed. Anthropometry was performed on all children after the correction of dehydration. All findings were recorded in a case record form.

Collection and processing of stool samples

A freshly passed stool sample was collected in a wide-mouthed, clean container. A part of the stool sample was concentrated by the formal ether sedimentation method for wet-mount microscopy for ova, parasites, and cysts. The second part of the sample was enriched in selenite F broth (SFB) and alkaline peptone water (APW) for four to six hours at 37º C and cultured on MacConkey’s medium, xylose lysine deoxycholate (XLD) medium, and bile salt agar (BSA) medium for the isolation of pathogenic enteric bacteria by standard laboratory methods. Antibiotic sensitivity testing of isolates was performed as per Clinical and Laboratory Standards Institute (CLSI) guidelines [7]. The third part of the sample was stored at -80 °C for PCR. The DNA was extracted from a stool sample using the QIAamp DNA Stool Mini Kit (QIAGEN, Hilden, Germany) and stored at -20 °C. The extracted DNA was subjected to PCR amplification using specific primers for the invasion plasmid antigen H (ipaH) gene at 424 bp for *Shigella *[8]. Forward 5’- GCTGGAAAAACTCAGTGCCT-3’ and reverse 5’- CCAGTCCTGTAAATTCATTCT-3’ amplification was performed using a reaction mixture of Taq polymerase, deoxynucleotide triphosphate (dNTP), and buffer (QIAGEN). Thirty-five amplification cycles were performed with the following steps: Initial denaturation at 95 °C for five minutes, cycling denaturation at 95 °C for 30 seconds, annealing at 56 °C for 30 seconds, extension at 72 °C for 40 seconds, and final extension at 72 °C for 10 minutes. The PCR amplicons were loaded onto a 2% agarose gel in a specific order for electrophoresis and run at 150 volts for 20-25 minutes. After electrophoresis, the 0.5 mM ethidium bromide-stained gel was visualized and interpreted for specific amplification patterns on the UV transilluminator.

Patient management

Hydration status was assessed, classified, and managed with ORS and intravenous fluids as per WHO guidelines [9]. Oral zinc was administered (10 mg/day for two- to six-month-olds, 20 mg/day for six- to 59-month-olds) for a total duration of 14 days. All children with visible blood in their stools received antimicrobial therapy as per WHO and Indian Academy of Pediatrics (IAP) guidelines [9, 10]. Children with bloody diarrhea, aged more than one year, with no risk factors (no malnutrition, no dehydration, preserved appetite) received oral antibiotics (cefixime), while those with malnutrition, severe dehydration, or poor oral acceptance received injectable antibiotics. Enrolled children were evaluated for comorbidities like hypoglycemia, hypothermia, and electrolyte imbalance and managed accordingly. Children with severe acute malnutrition (SAM) were stabilized and managed as per WHO guidelines [11].

Outcome variables

Outcomes were assessed as a percentage of children with a diagnosis of *Shigella *infection by molecular (PCR) and conventional methods (stool microscopy, culture). Children were discharged once the dehydration was corrected, stool frequency was reduced, and oral acceptance improved. After discharge, follow-up for persistence or recurrence of diarrhea was done on days three and seven, and then fortnightly for three months with simultaneous evaluation for growth faltering. Any recurrence of diarrhea or dysentery over three months after discharge was noted. The need for follow-up was emphasized by personal communication at each visit or by telephonic communication to minimize the attrition rate during follow-up.

Sample size

In an earlier study [12], *Shigella *infection was diagnosed by molecular methods in 11 out of 60 children with acute watery diarrhea (18.33%) and 34 out of 60 children with dysentery (56.66%). Assuming 10% of our enrolled cases to have dysentery (visible blood in stools), the estimated proportion of *Shigella *positivity by molecular methods was 22.2%. With an estimated proportion of 22.2%, the calculated sample size was 150 children with a relative precision of 30% (absolute precision of 6.66%) at a 95% confidence level. Thus, we planned to enroll 150 children with acute diarrhea.

Statistical analysis

Data collected were entered in a Microsoft Excel spreadsheet (Microsoft Corp., Redmond, WA) and analyzed using IBM Statistical Package for the Social Sciences (SPSS) software version 25 (IBM Corp., Armonk, NY). Outcome parameters such as frequency of isolation, duration of diarrhea, and recurrence of diarrhea were measured using descriptive statistics. Categorical variables were compared between *Shigella*-positive and *Shigella*-negative cases by the chi-square test to identify clinical characteristics and the outcome of *Shigella *infection. For normally distributed data, continuous variables between the two groups were compared using the Student's t-test, and for data that were not normally distributed, the Mann-Whitney U test was used. Odds ratios and 95% CI were calculated to determine risk factors (e.g., age < one month, socioeconomic status, malnutrition status) and clinical predictors (e.g., blood in stools, vomiting, dehydration) by univariate analysis.

## Results

Enrollment and baseline characteristics

**Figure 1:**
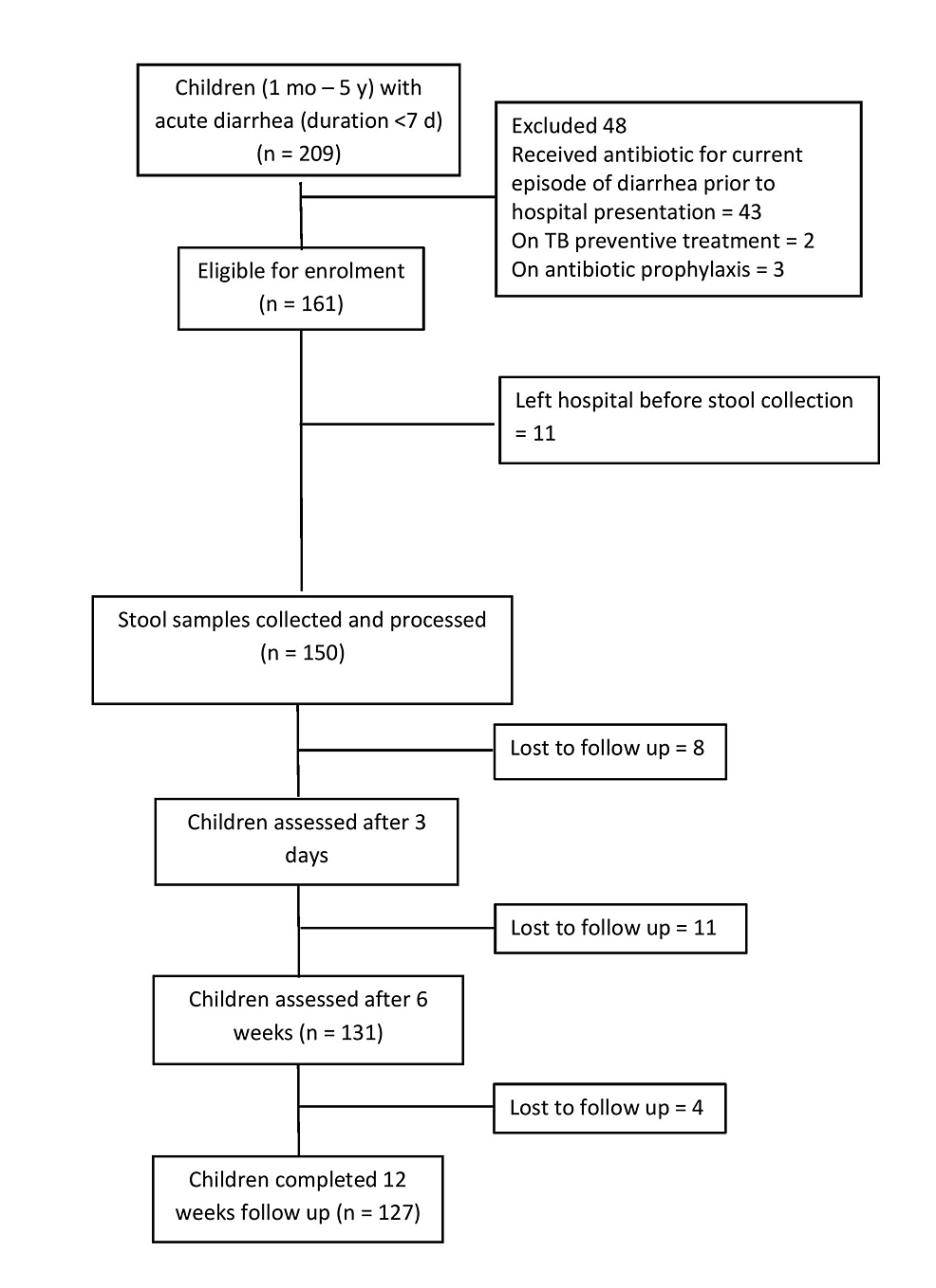
Flowchart depicting the selection of participants for the study mo: month; y: year; d: days; TB: tuberculosis

During the study period, 209 children aged between one month and five years with acute diarrhea were hospitalized, out of which 150 were included in the present study. Figure 1 depicts the flow of participants in the study, along with the reasons for the exclusion of cases not enrolled in the study.

The median (interquartile range (IQR)) age of enrolled children (72 (48%) males and 78 (52%) females) was 10 (5-23) months; 87 (58%) were infants (age <1 year). Only 94 (63%) were completely immunized for age. The majority (142, 94.7%) of the children’s families belonged to the lower middle, upper lower, or lower socioeconomic class on the Kuppuswamy scale [13]. The median (IQR) duration of diarrhea at hospitalization was two (one to three) days, and the number of stools passed in the previous 24 hours was 15 (10-20). Out of 150 children, 141 (94%) had watery, mucoid, or loose stools, and nine (6%) had blood in their stools. The most common associated symptom with diarrhea was vomiting (85%), followed by decreased oral acceptance (80%), and fever (40%).

Clinical characteristics

Undernutrition was prevalent in the enrolled children; 58 (38.7%) were stunted (height-for-age Z-score (HAZ) <−2SD) and 64 (42.7%) were wasted (weight-for-height Z-score (WHZ) <−2SD). Among these, 28 (19%) were severely stunted (HAZ <−3SD), while 40 (26.7%) children had SAM (weight for height Z-score <-3SD or mid-upper arm circumference <11.5 cm for children aged between six months and five years). At admission, dehydration was present in 138 (92%) children; 89 (59%) were severely dehydrated, and 15 (10%) had features suggestive of circulatory shock. All enrolled children received ORS during the hospitalization with a mean (SD) duration of 89.0 (31.3) hours, while IV fluids were administered to 95% of children for a mean (SD) duration of 44.1 (31.6) hours. Antibiotics were used in 101 (67.3%) cases for a mean (SD) of 5.3 (2.8) days. The presence of SAM (37%) and suspected or confirmed sepsis (33%) were the most common indications for the use of antibiotics in children admitted with acute diarrhea, followed by clinically suspected cholera (22%), dysentery (9%), pneumonia (5%), and meningitis (2%).

Stool microscopy

*Shigella sonnei* was diagnosed by stool culture in only one child. *Escherichia coli* was cultured from 67 stool samples, whereas *Klebsiella pneumonia*, trophozoites of *Giardia lamblia* and ova of *Ascaris lumbricoides*, and motile *Vibrio cholera* in hanging drop were detected in one stool sample each. *Shigella *was detected in 13 (8.7%) out of 150 stool samples by PCR amplification using the ipaH gene. The only culture-positive case was also detected by PCR. The sensitivity of stool culture was 7.7% against PCR for the diagnosis of *Shigella *infection. Out of 13 children in whom the *Shigella *PCR was positive, 11 (84.6%) had non-bloody diarrhea, and only two (15.4%) had dysentery. In 11 children with *Shigella *PCR positivity and non-bloody diarrhea, the characteristics of the stool did not change to bloody diarrhea until the resolution of the diarrhea episode, but eight of these children had received antibiotics due to associated comorbidities. Among three children with PCR positivity and non-bloody diarrhea managed without antibiotics, two had vomiting and poor oral acceptance at presentation, whereas none of them had fever or abdominal pain. All three children presented with some or severe dehydration, and none of these children developed bloody diarrhea or persistent diarrhea, despite no antibiotic usage.

Comparison of the characteristics and outcomes of children who were PCR-positive vs. PCR-negative for *Shigella*

Table 1 compares the clinical and anthropometric characteristics of children with stool PCR positivity for *Shigella *with those who had a negative test result on PCR.

**Table 1:** Comparison of clinical and anthropometric parameters at presentation between children with or without stool PCR Shigella positivity ^a^Data in number (%) unless stated otherwise; ^b^n=7 missing cases as the measurement taken for children >6 months; ^c^n=136 as the value could not be calculated for children with a length less than 45 cm; ^d^n=96 missing cases as the measurement taken for children >6 months n: number of patients; IQR: interquartile range; SD: standard deviation; PCR: polymerase chain reaction; SAM: severe acute malnutrition

Parameter	Stool PCR-positive (n=13, 8.7%)^a^	Stool PCR-negative (n=137, 91.3%)^a^	P-value
Diarrhea duration (days); median (IQR)	2.00 (1.0, 3.0)	2.00 (1.0,3.0)	0.908
No. of stools in last 24 hours; median (IQR)	15.00 (8.0, 19.0)	15.00 (11.0, 20.0)	0.390
Blood in stools	2 (15.4%)	7 (5.1%)	0.176
Vomiting	11 (84.6%)	116 (84.7%)	0.996
Fever	6 (46.2%)	54 (39.4%)	0.636
Abdominal pain	0	8 (5.8%)	1.000
Poor oral acceptance	10 (76.9%)	89 (65%)	0.544
Convulsions	1 (7.7%)	8 (5.8%)	0.568
Rash	0	5 (3.6%)	1.000
Cough	1 (7.7%)	18 (13.1%)	1.000
Immunized for age	7 (53.8%)	87 (63.5%)	0.679
Dehydration	11 (84.6%)	127 (92.7%)	0.465
Anthropometry at enrolment			
Weight for age (WFA) (kg); mean ± SD	6.62 (2.30)	6.76 (2.68)	0.847
Height for age (HFA) (cm); mean ± SD	69.10 (12.91)	71.05 (13.63)	0.620
Weight for age Z-score (WAZ); mean ± SD	-2.62 (1.60)	-2.98 (1.35)	0.359
Height for age Z-score (HAZ); mean ± SD	-1.62 (1.63)	-1.79 (1.69)	0.724
Weight for height Z-score (WHZ); mean ± SD	-2.10 (1.97)	-2.68 (1.58)^ c^	0.219
Mid upper arm circumference (MUAC) (cm); mean ± SD	12.36 (1.53)^ b^	12.48 (1.21)^ d^	0.803
Wasting	2 (15.4%)	31 (22.8%)	0.607
Severe wasting	4 (30.8%)	27 (19.9%) ^c^	-
Stunting	2 (15.4%)	28 (19%)	0.890
Severe stunting	3 (23.1%)	25 (18.3%)	-
SAM	5 (38.5%)	35 (25.7%)	0.336

There was no statistically significant difference between the two groups. The mean (SD) duration of diarrhea, as monitored after enrolment, was not significantly different (P=0.94) between PCR-positive participants (2.2 (1.54) days) and PCR-negative participants (2.3 (1.45) days). On univariate analysis, none of the evaluated risk factors or clinical predictors had a significant association with the *Shigella *infection (Table 2).

**Table 2:** Analysis of risk factors and clinical predictors of Shigella infection n: number of patients; PCR: polymerase chain reaction; SAM: severe acute malnutrition.

Parameter	Stool PCR-positive cases (n=13, 8.7%)	Stool PCR-negative cases (n=137, 91.3%)	Odds ratio (95% CI)
Age <1 year	9 (69.2%)	78 (56.9%)	1.70 (0.50-5.80)
Male gender	7 (53.8%)	65 (47.4%)	1.29 (0.41-4.04)
Lower socioeconomic status	12 (92.3%)	130 (94.9%)	0.65 (0.07-5.70)
Blood in stools	2 (15.4%)	7 (5.1%)	3.38 (0.62-18.26)
Vomiting	11 (84.6%)	116 (84.7%)	1.00 (0.21-4.82)
Poor oral acceptance	10 (76.9%)	89 (65%)	1.8 (0.47-6.85)
Convulsion	1 (7.7%)	8 (5.8%)	1.34 (0.15-11.67)
Cough	1 (7.7%)	18 (13.1%)	0.55 (0.07-4.50)
Dehydration	11 (84.6%)	127 (92.7%)	0.43 (0.08-2.23)
Wasting	6 (46.2%)	58 (42.3%)	1.17 (0.37-3.66)
Stunting	5 (38.5%)	51 (37.2%)	1.05 (0.33-3.40)
SAM	5 (38.5%)	35 (25.7%)	1.82 (0.56-5.94)

Follow-up

Out of 150 enrolled children, 23 were lost to follow-up, whereas 43 had a recurrence of diarrhea over the next three months. Among these, 14 patients required hospitalization, and antibiotics were used in 12 patients. During follow-up, only two patients had dysentery, and one patient developed persistent diarrhea. The recurrence of diarrhea episodes over three months was seen slightly more often among the children who had initial PCR (5, 45.4%) positivity than in PCR-negative participants (38, 37.8%), but this was not statistically significant (P=0.507). Also, there were no statistically significant differences in outcome in terms of growth faltering, recurrence of diarrhea, or hospitalization (Table 3).

**Table 3:** Outcome of enrolled children with acute diarrhea at the three-month follow-up ^a^n=127 (11 stool PCR-positive cases, 116 stool PCR-negative cases); ^b^n=43 (5 stool PCR-positive cases, 38 stool PCR-negative cases) PCR: polymerase chain reaction Binomial analysis was not done for persistent diarrhea, bloody diarrhea, and mortality as none of the PCR cases who completed the three months of follow-up had any of these outcomes.

Parameter	Stool PCR-positive cases (n=13, 8.7%)	Stool PCR-negative cases (n=137, 91.3%)	Odds ratio (95% CI)	P-value
Duration of diarrhea ≥7 days	4/13 (30.8)	50 (36.1)	0.77 (0.23-2.64)	0.68
Recurrence in the next 3 months^ a^	5 (45.5)	38 (32.8)	1.71 (0.49-5.96)	0.51
Hospitalization in the next 3 months ^b^	1 (20)	13 (34.2)	0.48 (0.05-4.75)	1.00

## Discussion

In this hospital-based study on under-five children with acute diarrhea, we documented that stool culture's diagnostic yield was very low compared to stool PCR amplification using a specific primer for the ipaH gene for the diagnosis of *Shigella *infection. The majority of *Shigella *infections presented with watery diarrhea rather than bloody diarrhea, and a history of blood in stools was a poor marker for the diagnosis of shigellosis. We documented that the molecular diagnostic approach performed better than conventional culture methods for the diagnosis of shigellosis. We could not find any significant clinical predictors associated with *Shigella *infection. There was no statistically significant difference in the overall outcome between PCR-positive and PCR-negative children at three months of follow-up.

The conventional methods of detection of *Shigella *infection rely on the stool culture, which turns out positive in a small fraction of actual cases of shigellosis due to multiple reasons like low bacterial load, loss of bacterial viability due to changes in ambient temperature and pH during specimen transport and storage, and use of antibiotics before specimen collection, transport, and storage [14]. Molecular diagnostic methods seem to overcome some of the shortcomings of conventional diagnostic methods, thus improving the diagnosis of *Shigella *infection. The use of PCR assays based on the amplification of the ipaH gene sequence is one such molecular method for diagnosis in cases of dysentery, as it is carried by all four species of *Shigella *[15].

Recent data from other settings also suggest that conventional culture yield is poor in episodes of *Shigella* diarrhea in young children [16]. The PCR-derived incidence for *Shigella *surpassed the original estimates by two-fold in the GEMS re-analysis, thus making it the most attributable pathogen of diarrhea among children under five [5]. In a re-analysis by PCR of stool samples from the multi-country Malnutrition and Enteric Disease (MAL-ED) Study, *Shigella*-attributable incidence increased from 4% to 41.3% in children aged between 12-24 months [17]. Earlier, Dutta et al. from Kolkata, India, reported a *Shigella *detection rate of 15.3% (46 of 300) by stool PCR as against 7.7% (23 of 300) by stool culture [8]. The positivity rate in the study by Dutta et al. was higher than that seen in our study, which is likely to be due to the inclusion of a very high proportion (42%; 126 out of 300) of children with dysentery. Similarly, Aggarwal et al. reported that the prevalence of *Shigella *increased from 8% (17 of 207) to 18.3% (11 of 60) in diarrhea and from 33% (39 of 118) to 56.7% (34 of 60) in dysentery when stool samples were analyzed by culture and PCR, respectively [12].

In two large population-based surveillance studies from Vietnam and China, an ipaH gene amplification-based PCR assay was used to detect *Shigella spp*. from the rectal swab specimens of cases presenting with dysentery. Results showed the presence of the ipaH gene in approximately 93%-97% of randomly selected *Shigella *culture-positive specimens and 46%-58% of randomly selected *Shigella *culture-negative specimens [18, 19]. In a study by Von Seidlein et al. on the analysis of pooled data from studies from six Southeast Asian countries, i.e., Bangladesh, China, Pakistan, Indonesia, Vietnam, and Thailand, *Shigella *was isolated from 2,927 (5%) of 56,958 diarrhea episodes, and more than half of these were in children under the age of five, and PCR detected the ipaH gene in 33% of samples of culture-negative stool specimens [20]. In a study from Bangladesh, Islam et al. documented that a PCR assay based on an ipaH probe improved the rate of detection of *Shigella *in stool samples to 61% from 44% by the conventional culture method [21]. They also tested 123 strains of *E. coli* by PCR to identify enteroinvasive *E. coli*, but none yielded any positive results. Though these studies have included variable age groups, the high detection rates of the ipaH gene in culture-negative stool specimens suggest that earlier estimates of shigellosis burden measured by conventional culture may have underestimated the true disease burden.

Blood in stools has been used as a surrogate marker for *Shigella *diarrhea. The WHO's diarrhea management guidelines recommend antibiotics effective against *Shigella *only when visible blood is present in stools. In our study, 84.6% (11 of 13) of the children with stool PCR positivity for *Shigella *presented with non-bloody diarrhea. In the GEMS re-analysis, *Shigella spp*. was the second highest cause of watery diarrhea, and overall, 40.3% (~527 of 1,310) of cases due to S*higella spp.* were non-dysenteric [5]. Likewise, 86.2% of the attributable incidence for *Shigella *was non-dysenteric in the re-analysis of the MAL-ED cohort study [17]. The results of a recent meta-analysis showed that the proportion of *Shigella *infections presenting as dysentery has been decreasing [22]. This may be related to the diagnosis of such cases by molecular methods or early treatment with antibiotics. However, it is unclear whether *Shigella *infection without blood in stools needs therapy on similar lines. In our series of cases, antibiotics were administered for other reasons in cases where *Shigella *was later detected by molecular methods, except in three children in whom the infection resolved without any use of antibiotics.

The strength of our study was the prospective nature of enrollment, with a focus on the clinical presentation and outcome of *Shigella *infection detected with molecular methods. Our study also attempted to evaluate the clinical profile (resolution of diarrhea, change to bloody diarrhea in those with watery diarrhea, and recurrence) during a three-month follow-up period for children with *Shigella *infection managed as per current diarrhea treatment guidelines, which has not been evaluated in any of the studies comparing detection rates of *Shigella *between conventional and molecular methods. However, because of antibiotic use for other indications, we were unable to validly comment on the outcome of PCR-positive cases when they are managed without antibiotics. A relatively low *Shigella *prevalence in our series precluded the identification of any significant clinical marker of *Shigella *infection. Another limitation of our study was the use of the ipaH gene as a marker for *Shigella *detection, which may also be found in enteroinvasive *E. coli*. We enrolled hospitalized patients who primarily had moderate-to-severe diarrhea and concurrent illnesses; therefore, results from our study may not be generalizable to community settings with less severe diarrheal illnesses.

## Conclusions

The present study concludes that *Shigella *is an important cause of diarrhea in children under five, often missed by conventional laboratory methods. Blood in stools as a syndromic indicator for *Shigella *infection needs to be reconsidered as the majority of *Shigella *diarrhea cases have non-bloody stools. As the current management guidelines for childhood diarrhea recommend antibiotics for only bloody diarrhea, studies evaluating other clinical predictors of *Shigella *infection and faster and more cost-effective techniques for its molecular diagnosis are required. Large, community-based longitudinal studies are needed to evaluate the outcome of non-dysenteric *Shigella *infections diagnosed by molecular methods so that management guidelines for such infections can be formulated.
